# Long‐term outcome in a case of translocated mitral valve replacement for massive mitral annular calcification

**DOI:** 10.1002/ccr3.862

**Published:** 2017-03-02

**Authors:** Go Kataoka, Kiyoharu Nakano, Ryota Asano, Atsuhiko Sato, Wataru Tatsuishi

**Affiliations:** ^1^ Department of Cardiovascular SurgeryTokyo Women's Medical University, Medical Center EastTokyoJapan

**Keywords:** Calcification, dialysis, mitral stenosis

## Abstract

Few reports have described long‐term outcomes after translocated mitral valve replacement. We describe tips, potential pitfalls, and long‐term outcome associated with the construction of a new mitral annulus and reinforcement of prosthesis attachment using a mitral prosthetic valve with an equine pericardial collar in a woman with extensive mitral valve calcification.

## Background

Calcification of the mitral valve (MV) annulus is an age‐related degenerative process that involves the posterior aspect of the annulus, and it can spread to the atrial and ventricular walls 1. Severe calcification of the MV annulus is often observed in patients undergoing long‐term hemodialysis. In most cases of MV repair among patients with extensive calcification of the mitral annulus, calcification prevents appropriate insertion of the prosthetic valve, and it may increase the frequency of postoperative periprosthetic leakage or valvular dehiscence 2. Moreover, MV replacement (MVR) in this setting may be associated with a high risk of fatal complications such as left ventricular rupture, coronary circumflex artery injury, and thromboembolic events 1. Furthermore, long‐term durability after translocated MVR is unknown. We performed modified translocated MVR in a patient with extensive annular calcification of the MV to reduce the occurrence of fatal operative complications and postoperative periprosthetic leakage or valvular dehiscence for improved long‐term outcomes.

## Case Report

**Figure 1:**
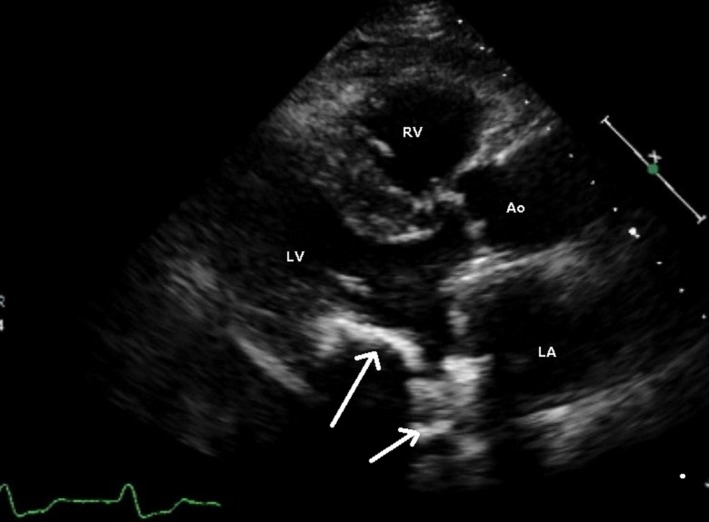
Preoperative transthoracic echocardiogram. Extended calcification of the posterior leaflet of the mitral annulus, part of the left ventricle, and papillary muscles (white arrows) is shown. Ao, aorta; LA, left atrium; LV, left ventricle; RV, right ventricle.

A 55‐year‐old woman on hemodialysis for chronic glomerular nephritis for 18 years had undergone dual‐chamber pacemaker implantation because of a complete atrioventricular block 4 years before presentation. The preoperative transthoracic echocardiogram indicated severe mitral stenosis with an estimated pressure gradient of 15 mmHg and a MV area of 0.8 cm^2^. In particular, the posterior leaflet of the mitral annulus, a part of the left ventricle, and papillary muscles were severely calcified (Fig. 1). The preoperative computed tomography scan demonstrated severe calcification around the mitral annulus and calcification invading the left ventricle. Cardiac catheterization showed normal coronary arteries with normal left ventricular function (ejection fraction, 70%).

**Figure 2:**
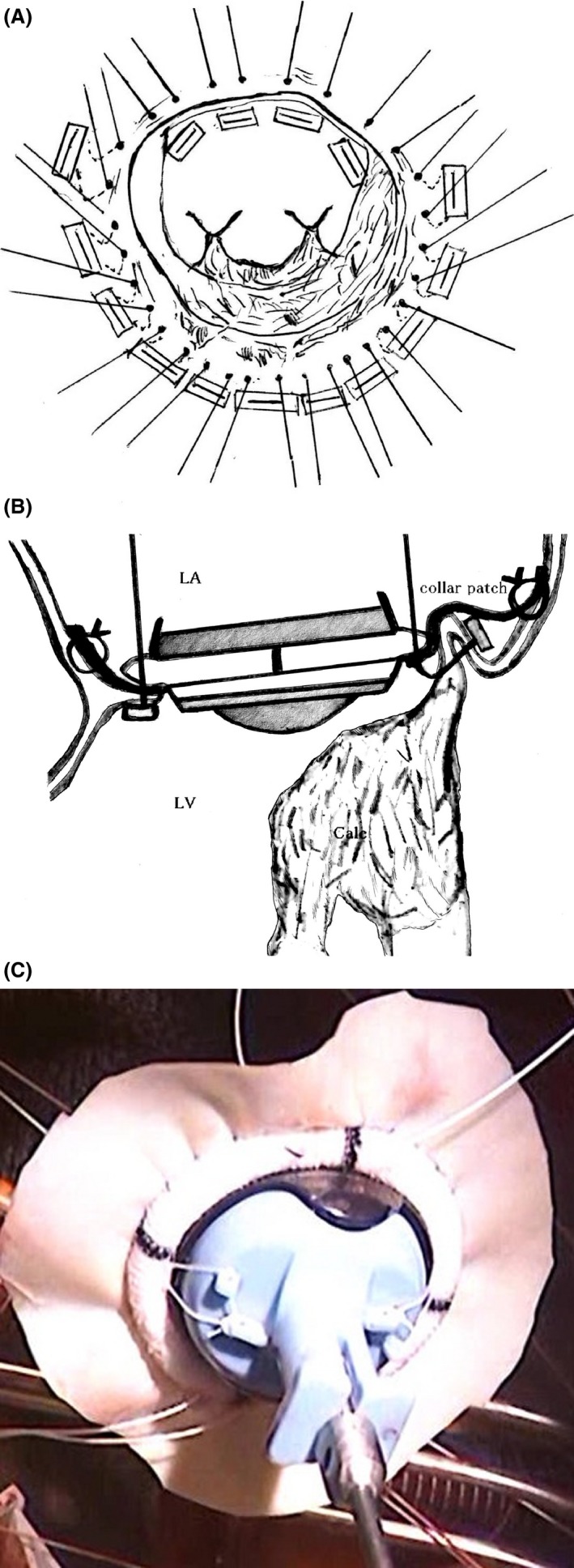
(A) A 29‐mm SJM valve is secured in the supraannular position of the same plane. (B) Mitral valve replacement. Four sutures are placed in a supraannular manner on the anterior mitral annulus, and an additional 11 sutures are placed through all the layers of the left atrial wall. (C) A 1.5‐cm wide equine pericardial collar sutured to the ring of a 29‐mm SJM valve. Calc, calcification; LA, left atrium; LV, left ventricle; SJM, St. Jude Medical.

The operation was performed through median sternotomy. Cardiopulmonary bypass was established through ascending aortic cannulation and bicaval drainage. A left venting tube was inserted through the right superior pulmonary vein. After cardiac arrest, the right side of the left atrium was incised. The tissue surrounding the mitral annulus, especially the posterior mitral annulus, posterior leaflet, and part of the anterior leaflet, including the chordae, were severely calcified. The posterior leaflet and part of the anterior leaflet could not be excised because of extensive calcification. For MVR, four sutures were placed at the supraannular position on the anterior mitral annulus. Eleven additional sutures were placed through all the layers of the left atrial walls so that they wrapped around the posterior mitral annulus (Fig. 2A and B). A 29‐mm expanded cuff of a mitral prosthetic valve (St. Jude Medical [SJM], Inc., St. Paul, MN) was enlarged at the circumference of the suture ring by attaching a 1.5‐cm wide equine pericardial collar (Equine Pericardial Patch; Edwards Lifesciences, LLC, Irvine, CA; Fig. 2C); this cuff was secured with 2‐0 polyester pledgeted mattress sutures. The free edge of the equine pericardial collar was sutured to the atrial wall with a running 4‐0 polypropylene suture for additional reinforcement. The mechanical valve was placed in anti‐anatomical position. The patient was weaned off cardiopulmonary support uneventfully. The aortic cross clamp time, cardiopulmonary bypass time, and operative time were 146, 176 and 311 min, respectively. The postprocedural transesophageal echocardiogram showed no periprosthetic leakage.

**Figure 3:**
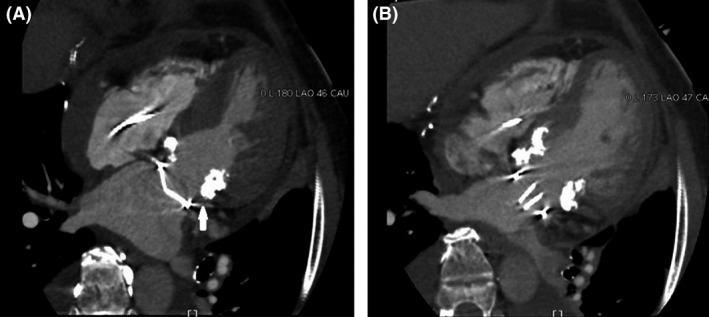
(A) Postoperative multidetector computed tomography scan of the left ventricle during the systolic phase. Adequate closure of the SJM valve is shown. The white arrow indicates that the SJM valve was displaced from the posterior mitral annulus with extensive calcification to the left atrium. (B) Postoperative multidetector computed tomography scan of the left ventricle during the diastolic phase. Adequate opening of the SJM valve is shown. The SJM valve is not interrupted by the subannular apparatus. SJM, St. Jude Medical.

The postoperative multidetector computed tomography scan demonstrated good opening and closing of the prosthetic valve during the systolic and diastolic phases (Fig. 3A and B). The patient's postoperative course was uneventful, and she was discharged on the fourteenth postoperative day. She had satisfactory improvement, and the transthoracic echocardiogram showed normal prosthetic valve function without periprosthetic leakage and a mean transvalvular pressure gradient of 6 mmHg at 45 months postoperatively.

## Discussion

In patients undergoing dialysis, mechanical stress caused by hypertension and a high cardiac output due to an arteriovenous shunt can lead to the development of mitral annular calcification, which in some cases may extend to the valve leaflets and myocardium 3. Several surgical techniques have been used to manage extensive calcification at the mitral annulus, such as the active removal of calcification and posterior mitral annular reconstruction using an anterior MV patch 4, 5 to create a new annulus 6, 7, or a combination of these techniques 2. Complete calcium debridement may cause atrioventricular rupture, left circumflex coronary artery damage, and/or thromboembolic events 1. Intra‐atrial insertion of the prosthesis after constructing a new annulus may lead to severe hemorrhage or valve dehiscence due to the transfer of high left ventricular pressure into the left atrium 8.

To prevent fatal complications in the present patient, we did not remove the posterior mitral leaflet and part of the anterior mitral leaflet because of extensive calcification. The technique of avoiding active removal of severe calcification was considered reliable and reproducible. We propose the following three approaches during modified translocated MVR to reduce complications. First, all needles should exit in the same arterial plane, as this enables the prosthesis to be placed in the supraannular position, thus reducing periprosthetic leakage. MVR performed in the supraannular position enables surgeons to insert a larger prosthesis. Second, the sutures should be placed through all the layers of each of the two atrial walls so that they wrap around the posterior mitral annulus. In addition, the mitral prosthetic valve with an equine pericardial collar should be sutured using a running 4‐0 polypropylene suture to reinforce the attachment of the prosthetic valve. Finally, the SJM expanded cuff of the mitral prosthetic valve should be used, as it increases the contact area between the sutured cuff and atrium and prevents disturbance of the opening of the prosthetic leaflets caused by the remaining extensively calcified native posterior leaflet. SJM valves have the advantage of causing minimal interference between the leaflets and subvalvular tissue because the height of this valve leaflet from the support frame is the lowest (3.4 mm) among other mitral mechanical prostheses 9. Kato et al. reported that a paravalvular leak occurred in one of five patients who had undergone MVR by simple supraannular prosthesis insertion 10. From their viewpoint, sutures of the sewing cuff, both layers of the left atrial wall, and collar patch are very important to reduce the chance of a paravalvular leak. However, surgeons need to pay attention to avoid involving the left circumflex artery when placing sutures on the posterior side of the left atrial wall.

We consider that using these techniques, translocated MVR can reduce the perioperative risk and limit reintervention after MVR in a patient with extensive calcification of the mitral annulus, especially when the calcified posterior mitral leaflet cannot be excised. We believe that our techniques may be suitable for performing MVR in patients with extensive calcification of the mitral annulus, and good long‐term outcomes may be obtained.

## Consent

Written informed consent was obtained from the patient for publication of this case report and any accompanying images. A copy of the written consent is available for review by the editor in chief of this journal.

## Authorship

GK: made substantial contributions to the conception and design, acquisition of data, and analysis and interpretation of data. KN: was involved in drafting the manuscript or revising it critically for important intellectual content. RA, AS, and WT: approved the final version to be published. All authors read and approved the final manuscript.

## Conflict of Interest

The authors declare no conflict of interest.

## References

[1] Giovanni, R. , G. Speziale , P. Voci , and B. Marino . 1997 “Patch‐glue” annular reconstruction for mitral valve replacement in severely calcified mitral annulus. Ann. Thorac. Surg. 63:570–571. 9033351 10.1016/s0003-4975(96)01025-9

[2] Bito, Y. , T. Shibata , T. Yasuoka , K. Inoue , and T. Ikuta . 2008 Mitral valve replacement for extensive calcification: half and half technique. Gen. Thorac. Cardiovasc. Surg. 56:526–528. 18854933 10.1007/s11748-008-0296-x

[3] Iida, H. , Y. Mochizuki , Y. Matsushita , H. Mori , Y. Yamada , and S. Miyoshi . 2005 A valve replacement technique for heavy calcified mitral valve and annulus. J. Heart Valve Dis. 14:209–211. 15792181

[4] Nezic, D. , A. Knezevic , S. Borovic , and M. Jovic . 2012 Mitral valve replacement with posterior transposition of the anterior leaflet which covers and buttresses partially decalcified posterior mitral annular bed. Eur. J. Cardiothorac. Surg. 41:1129–1131. 22219486 10.1093/ejcts/ezr166

[5] David, T. E. , C. M. Feindel , S. Armstrong , and Z. Sun . 1995 Reconstruction of the mitral annulus. A ten‐year experience. J. Thorac. Cardiovasc. Surg. 110:1323–1332. 7475184 10.1016/S0022-5223(95)70055-2

[6] Di Stefano, S. , J. López , S. Flórez , J. Rey , A. Arevalo , and A. San Román . 2009 Building a new annulus: a technique for mitral valve replacement in heavily calcified annulus. Ann. Thorac. Surg. 87:1625–1627. 19379934 10.1016/j.athoracsur.2008.09.014

[7] Atoui, R. , V. Lash , S. Mohammadi , and R. Cecere . 2009 Intra‐atrial implantation of a mitral valve prosthesis in a heavily calcified mitral annulus. Eur. J. Cardiothorac. Surg. 36:776–778. 19632128 10.1016/j.ejcts.2009.05.035

[8] Fukuda, Y. , Y. Matsui , Y. Bito , S. Sasaki , and K. Yasuda . 2005 A case of mitral valve replacement with a collar‐reinforced prosthetic valve for heavily calcified mitral annulus. Ann. Thorac. Cardiovasc. Surg. 11:260–263. 16148875

[9] Kurazumi, H. , A. Mikamo , R. Suzuki , and K. Hamano . 2011 Mitral‐valve replacement for a severely calcified mitral annulus: a simple and novel technique. Eur. J. Cardiothorac. Surg. 39:407–409. 20696586 10.1016/j.ejcts.2010.06.017

[10] Kato, Y. , K. Hattori , Y. Bito , S. Kotani , K. Inoue , and T. Shibata . 2011 Simple supra‐annular prosthesis insertion with extensive mitral annular calcification. J. Heart Valve Dis. 20:180–183. 21560819

